# ﻿A new species of *Struthanthus* Mart. (Loranthaceae) from Oaxaca, Mexico

**DOI:** 10.3897/phytokeys.225.101238

**Published:** 2023-04-21

**Authors:** Maria Guadalupe Maldonado-Borja, Rosa Cerros-Tlatilpa, Luis Gil Galván-González

**Affiliations:** 1 Maestría en Manejo de Recursos Naturales, Centro de Investigaciones Biológicas, Universidad Autónoma del Estado de Morelos, Av. Universidad 1001, Col. Chamilpa, Cuernavaca, Morelos, 62209, Mexico Universidad Autónoma del Estado de Morelos Cuernavaca Mexico; 2 Facultad de Ciencias Biológicas, Universidad Autónoma del Estado de Morelos, Av. Universidad 1001, Col. Chamilpa, Cuernavaca, Morelos, 62209, Mexico Universidad Autónoma del Estado de Morelos Cuernavaca Mexico

**Keywords:** Endemic, hemiparasitic, mistletoes, taxonomy

## Abstract

*Struthanthusibe-dzi***sp. nov.** is a new species described and illustrated from the cloud and pine-oak forests of the Sierra Madre del Sur in Oaxaca, Mexico. This species shares similarities of leaf shape and inflorescence type with *S.deppeanus*, *S.quercicola*, and *S.ramiro-cruzii*. However, *S.ibe-dzi* can be recognized by its glaucous branches, leaves and inflorescences; compressed nodes; convoluted distal half of styles in pistillate flowers; and staminate flowers with asymmetrical thecae and an extended connective forming an apiculate horn in both anther series. A distribution map and an identification key are provided to separate *S.ibe-dzi* from morphologically similar congeners present in the region.

## Abstract (de)

*Struthanthusibe-dzi***sp. nov.**, es una nueva especie descrita e ilustrada de los bosques de niebla y de pino-encino de la Sierra Madre del Sur en Oaxaca (México). La nueva especie se puede confundir con *S.deppeanus*, *S.quercicola*, y *S.ramiro-cruzii* por la forma de la hoja y el tipo de inflorescencia. Sin embargo, *S.ibe-dzi* se distingue por sus ramas, hojas e inflorescencias glaucas; sus nodos comprimidos; flores pistiladas con el estilo convoluto desde la mitad hacia la parte distal; y flores estaminadas con tecas asimétricas y un conectivo extendido apiculado en forma de cuerno en la serie baja de las anteras. Se incluye un mapa de distribución y una clave de identificación para separar *S.ibe-dzi* de sus congéneres regionales morfológicamente similares.

## ﻿Introduction

*Struthanthus* Mart. is a neotropical genus in the Loranthaceae family with approximately 60–70 species (Caires and Dettke 2020) distributed from Northern Mexico to Argentina (Kuijt and Hansen 2015). The genus comprises hemiparasitic aerial shrubs or climbing plants which are dioecious (Kuijt 2009a; Kuijt and Hansen 2015; Nickrent et al. 2019). *Struthanthus* is commonly recognized by the presence of thick epicortical roots at the base of the plant and along stems. Additional important characters include stems teretes, sub-teretes or quadrangular (when young), with lenticels and a glabrous, striated, or sulcate texture (Kuijt 2009a; Kuijt and Hansen 2015; Caires and Dettke 2020); leaves opposite, subopposite, or rarely alternate, sometimes with stiffly recurved leaves and prehensile petioles (Caraballo-Ortiz and Acevedo-Rodríguez 2021; Maldonado 2021); axillar and indeterminate inflorescences in racemes, spikes, or head-like with pedunculate or sessile triads; flowers small (4–10 mm long), hexamerous, pedicellate or sessile; staminate flowers with dimorphic stamens, dorsifixed (Kuijt 2009b; Kuijt and Hansen 2015) but basifixed in at least some of the Mexican species (Maldonado 2021); and pistillate flowers with monomorphic staminodes and styles straight to strongly convolute in some Mexican species (Kuijt 1975a, 2003a; Maldonado 2021).

Despite the lack of a comprehensive taxonomic treatment for *Struthanthus*, new species have been described during the last 20 years (Kuijt 2003a, b, 2009a, 2014; González and Morales 2004; Martínez-Ambriz et al. 2017). Also, several generic transfers have occurred, including *Cladocolea* Tiegh. (Kuijt 1987, 1991b), *Panamanthus* Kuijt (Kuijt 1991a), *Passovia* H. Karst. (Kuijt 2011), *Peristhetium* Tiegh. (Kuijt 2012), *Phthirusa* Mart. (Kuijt 2009a) and *Pusillanthus* Kuijt (Caires et al. 2012).

Regarding generic affinities, *Struthanthus* has been found to be closely related to *Cladocolea*. This relationship has been supported by pollen morphology as described in Grímsson et al. (2018), where both genera were found to share type B pollen grains, showing an affinity to *Peristethium*. Molecular sequence data also support a relationship between *Struthanthus* and *Cladocolea* based on plastid DNA sequences (*matK*, *rbcL*, and *trnL*-*F*) and ribosomal nuclear cistron (SSU rDNA and LSU rDNA) (Vidal-Russell and Nickrent 2008). However, Kuijt and Lye (2005) analyzed foliar sclerenchyma across multiple species of *Struthanthus* and *Cladocolea* and found a wide morphological diversity in both genera, suggesting that they are polyphyletic as currently defined. This idea is supported by surveys of additional morphological characters from multiple species of *Struthanthus*, where taxa exhibit a wide variation typically observed in distantly related lineages (Kuijt 1975b, 1981, 2012; Kuijt and Hansen 2015).

About 14 species of *Struthanthus* have been reported from Mexico (Nickrent 2020; Maldonado 2021), with five of them endemic to the country. In the state of Oaxaca, seven species have been documented: *S.capitatus* Lundell, *S.crassipes* (Oliv.) Eichler, *S.deppeanus* (Schltdl. & Cham.) Blume, *S.hartwegii* (Benth.) Standl., *S.interruptus* (Kunth) G.Don, *S.matudae* Lundell, and *S.quercicola* (Schltdl. & Cham.) Blume. Here we describe a new species of *Struthanthus* from Oaxaca that shares morphological affinities with *S.deppeanus*, *S.quercicola*, and *S.ramiro-cruzii* Martínez-Ambr. & Sor.-Benítez. These affinities are based on both vegetative (stems and leaf shape) and reproductive (raceme inflorescence) characters.

## ﻿Materials and methods

While preparing a floristic checklist for *Struthanthus* in Mexico, herbarium material and specimens collected during fieldwork were examined. The description provided below is based on our collections from Sierra Madre del Sur in the state of Oaxaca as well as herbarium specimens from ANSM, CHAPA, CIIDIR, ENCB, HUAA, HUAP, HUMO, IEB, MEXU, RSA, SLPM, UAMIZ, UAS, UAT, USON, XAL, and ZEA (acronyms follow Thiers (2021, continuously updated)).

Specimens were collected, pressed, and dried, with three to five duplicates per number. Vouchers were deposited at HUAP, HUMO, MEXU, and UAMIZ. All specimens gathered had reproductive structures (flowers and fruits) present. Morphological characters were measured from dried specimens and described using the terminology presented by Kuijt (1987, 2003a, b). Hosts and a distributional map are provided for *S.ibe-dzi*, as well as a taxonomic key and a comparative table to differentiate it from its three morphologically closest congeners in Mexico (*S.deppeanus*, *S.quercicola*, and *S.ramiro-cruzii*). The map was generated with QGIS (QGIS Development Team 2023) v 3.16. with points based on data from our field work and herbarium specimens from other collectors.

## ﻿Taxonomic treatment

### ﻿ Struthanthus ibe-dzi

Taxon classificationPlantaeSantalalesLoranthaceae

Mald. & Cerros sp. nov.

8FD291C4-77A7-5B33-93AB-4C7C9A6E0DE2

urn:lsid:ipni.org:names:77317963-1

#### Type.

**Mexico. Oaxaca**: Tlaxiaco, Río Ocotepec, en el arroyo Yute kuini (San Juan del Río Cuquila), carretera Tlaxiaco-Putla, 17°10'26.48"N, 97°46'17.29"W [17.174022°N, -97.771469°W], 1962 m a.s.l., 30 Mar 2021, *M.G. Maldonado, L.G. Galván G. & R. Cerros T. 21* (♀ fl, fr) (holotype: HUMO-39855!, isotypes: MEXU!, UAMIZ!).

#### Diagnosis.

*Struthanthusibe-dzi* morphologically resembles *S.deppeanus* and *S.quercicola* in having epicortical roots on stems, similar leaf shapes, and inflorescences in racemes. However, the new taxon differs by its compressed nodes, stems, leaves, and inflorescence glaucous; leaf blade with base cuneate to oblique; staminate flowers 6–9 mm long with asymmetrical thecae and an extended horn-shaped apiculate connective in both anther series; and pistillate flowers with distally convoluted styles.

#### Description.

**Figure 1.**
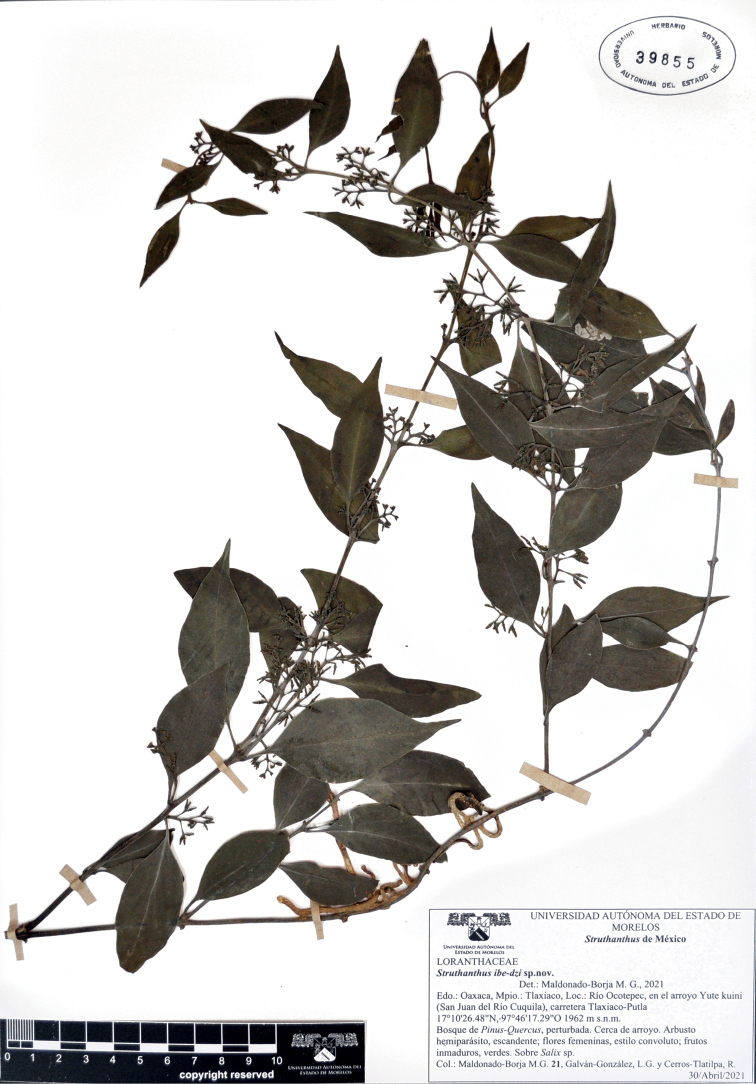
*Struthanthusibe-dzi* Mald. & Cerros, sp. nov. (Loranthaceae). Holotype: *M.G. Maldonado, L.G. Galván G. & R. Cerros T. 21* (♀ fl, fr), HUMO-39855.

**Figure 2.**
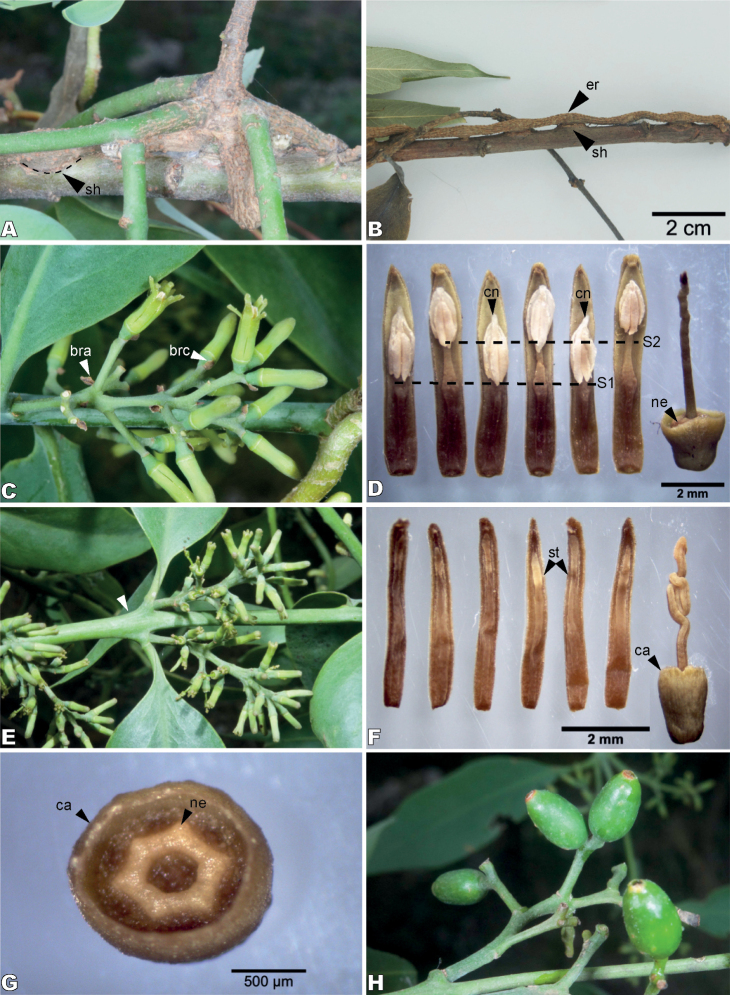
*Struthanthusibe-dzi* (Loranthaceae) **A** base of stem and disk of secondary haustoria (sh) **B** epicortical root (er) and swollen stem of host plant by the penetration of secondary haustoria (sh) **C** staminate inflorescences with caducous bracts (bra) and bracteoles (brc) **D** dissected petals of staminate flower showing two series of stamens (S1, S2), with anthers in the lower series (S1) displaying a prominent connectival horn (cn); receptacle showing an undifferentiated pistilloid and a nectary (ne) surrounded by the calyculus **E** pistillate inflorescences and compressed node (arrow) **F** dissected pistillate flower showing vestigial staminodes (st) opposite to petals, and an inferior ovary with convoluted style and calyculus (ca) **G** upper view of ovary in a pistillate flower showing its calyculus (ca) and nectary (ne) **H** immature fruits crowned by the calyculus.

Aerial hemiparasitic woody shrub, pendulous, perennial, with epicortical roots present at the base of main trunk; branches pendant. ***Stems*** green when young, brown with lenticels when mature; nodes glaucous, bicarinate and compressed, especially when young; internodes terete, with epicortical roots. ***Leaves*** opposite or subopposite; petioles 0.25–1.2 cm long, twisted, forming a shallow channel from the raised edge of leaf blade; blades ovate to lanceolate, rarely elliptical, 5.0–12.2 × 1.4–5.0 cm, papyraceous when dried; apex acute to acuminate, base cuneate to oblique, margin entire to repand, hyaline, venation pinnate. ***Inflorescences*** a solitary raceme of triads, indeterminate and axillar; bracts and bracteoles caducous at or after anthesis, cymbiform; rachis subterete to compressed, nodes compressed, triads opposite or subopposite, decussate, green, glabrous, glaucous. ***Staminate inflorescence*** 2.0–6.8 cm long, peduncle 0.2–0.8 cm with 6–16 (19) triads, triad peduncle 0.10–0.58 cm long. ***Pistillate inflorescence*** 2.0–5.0 cm long, peduncle 0.2–1.0 cm long with 6–12 (15) triads, triad peduncle 0.18–0.86 cm long. ***Staminate flowers*** hexamerous, rarely pentamerous, flower buds clavate with rounded apex; central flower of triad sessile, lateral pedicels 0.28–1.3 mm long; mature flowers 6.0–9.0 × 2.0–2.3 mm, petals linear, reflexed near the apex, 4.4–7.9 × 0.8–1.2 mm, anthers basifixed (not versatile) in two series, theca asymmetrical; prominent connectival apiculate horn in both the lower and upper series; calyculus irregularly dentate, whitish, vestigial ovary 1.5–2.2 mm; pistiloid straight to sigmoid 1/3 near the apex, 2.8–6.5 mm long, stigma undifferenced; nectary thick with six protuberances surrounding the pistiloid base. ***Pistillate flowers*** hexamerous, flower buds cylindrical, rounded at the apex; central flower of the triad sessile, pedicels of lateral flowers 0.18–0.8 cm long, slightly accrescent when bearing fruit; mature flowers 5.8–7.2 × 1.6–2.2 mm, linear petals 5.2–5.6 × 0.8–1.0 mm, staminodes in one series; calyculus whitish, irregularly dentate, inferior ovary 1.7–2.0 mm; style convolute 4.0–5.2 mm long (± 3 longitudinal folds) from the middle to the apex, stigma capitate; nectary thick with six protuberances surrounding the style. ***Fruit*** a one-seeded berry, ovoid, 3.85–6.0 × 6.52–8.60 mm. ***Seeds*** ovoid, 3.0–4.7 × 5.1–7.4 mm. Figs 1, 2.

#### Additional specimens examined

**(Paratypes). Mexico. Oaxaca**: San Juan Mixtepec, Yucu Shúun (Monte de Tesoro) a 16 km S de San Juan Mixtepec, 17°13'37.46"N, 97°47'54.5"W [17.22707°N, -97.78484°W], 2,500 m a.s.l., 8 Nov 1988 (♀ fr), *J. Reyes S. 1064* (MEXU, UC/JEPS); Putla Villa de Guerrero, 2.1 km después de Santo Domingo Chicahuaxtla, hacia Putla, [17.142761°N, -97.848589°W], 2,050 m a.s.l., 5 Feb 1993 (♀ fr), *M. Cházaro B. 7088* (CHAPA, ENCB, MEXU, XAL); Santiago Juxtlahuaca, El Manzanal, senda para la parcela del Sr. Hemeterio, entrada por Santa Rosa-San Miguel Cuevas, Distrito Juxtlahuaca, 17°13'13.20"N, 98°3'38.30"W [17.22033°N, -98.06063°W], 2,060 m a.s.l., 13 Sep 1996 (♀ fl, fr), *J.I. Calzada 21381* (MEXU); San Juan Mixtepec, Camino a Santos Reyes Tepejillo, 1 km antes de la desviación al Capulín y Tinuama de Zaragoza, 17°19'23.4"N, 97°54'13.2"W [17.32317°N, -97.90367°W], 2,650 m a.s.l., 9 Apr 2019 (♂ fl), *L.G. Galván G. & R. Cerros T. 474* (HUMO); Putla Villa de Guerrero, orilla de la carretera, km 92, 2.8 km antes de San Andres Chicahuaxtla, 17°10'48.20"N, 97°49'32.48"W [17.18014°N, -97.82569°W], 2,363 m a.s.l., 13 Feb 2020 (♀ fr), *M.G. Maldonado, R. Cerros T. & L.G. Galván G. 13* (HUMO); ibid, 13 Feb 2020 (♀ fr), *M.G. Maldonado, R. Cerros T. & L.G. Galván G. 14* (HUMO); Tlaxiaco, Río Ocotepec, en el arroyo Yute kuini (San Juan del Río Cuquila), carretera Tlaxiaco-Putla, 17°10'26.48"N, 97°46'17.29"W [17.17402°N, -97.77146°W], 1,962 m a.s.l., 30 Apr. 2021 (♂ fl), *M.G. Maldonado, R. Cerros T. & L.G. Galván G. 19* (HUMO); ibid, 30 Apr 2021(♀ fl), *M.G. Maldonado, R. Cerros T. & L.G. Galván G. 20* (HUMO); ibid, 30 Apr 2021 (♀ fl), *M.G. Maldonado, R. Cerros T. & L.G. Galván G. 22* (HUMO); ibid, 30 Apr 2021(♀ fl), *M.G. Maldonado, R. Cerros T. & L.G. Galván G. 23* (HUMO); Putla Villa de Guerrero, km 92, carretera Tlaxiaco-Putla, 200 m antes de La Cañada Tejocote, 17°10'48.38"N, 97°46'31.94"W [17.18010°N, -97.82554°W], 2,374 m a.s.l., 30 Apr 2021(♂ fl), *M.G. Maldonado, R. Cerros T. & L.G. Galván G. 24* (HUMO); ibid, 30 Apr 2021(♀ fl), *M.G. Maldonado, R. Cerros T. & L.G. Galván G. 25* (HUMO); ibid, 30 Apr 2021(♂ fl), *M.G. Maldonado, R. Cerros T. & L.G. Galván G. 26* (HUMO); Putla Villa de Guerrero, Orilla de carretera de Tlaxiaco-Putla, en San Andres Chicahuaxtla, 17°09'42.16"N, 97°50'11.85"W [17.16171°N, -97.83662°W], 2,473 m a.s.l., 30 Apr 2021(♂ fl), *M.G. Maldonado, R. Cerros T. & L.G. Galván G. 27* (HUMO).

#### Distribution, habitat, and hosts.

**Figure 3.**
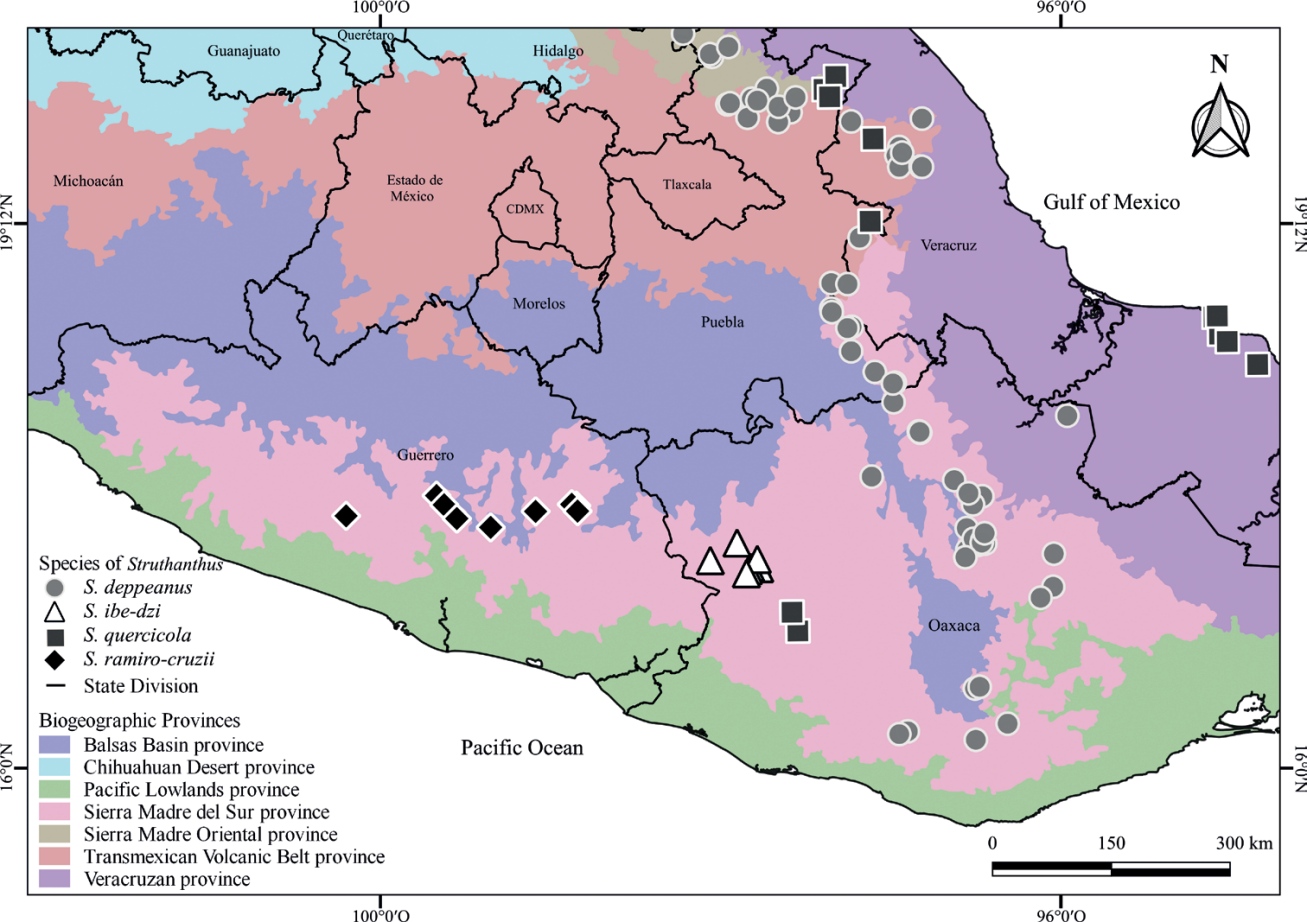
Distribution map of *Struthanthusdeppeanus*, *S.ibe-dzi*, *S.quercicola*, and *S.ramiro-cruzii* in Oaxaca and surrounding states of central Mexico.

*Struthanthusibe-dzi* is endemic to Oaxaca, Mexico, where it is only known from cloud and oak-pine forests with secondary vegetation in the Sierra Madre del Sur (Morrone et al. 2017) in the municipalities of Putla Villa de Guerrero, San Juan Mixtepec, Santiago Juxtlahuaca, and Tlaxiaco (Fig. 3) at elevations between 1,962 to 2,650 m a.s.l. Recorded hosts to date include *Alnus* spp. (Betulaceae), *Quercus* spp. (Fagaceae), and *Salix* spp. (Salicaceae).

#### Phenology.

Flowering from March to April and in September; fruiting in February to April and September to November. Individuals can be found bearing flowers and fruits on different branches.

#### Etymology.

The epithet *ibe-dzi* refers to the common name given to any mistletoe in Zapotec language (*Ibë-dzí*) in the San Juan Mixtepec region, which means “hair(s) on top of”, where “ibé” denotes “hair(s)” and “dzi” indicates “on top of”.

#### Conservation status.

*Struthanthusibe-dzi* is only known from the western part of the state of Oaxaca, near the border with the state of Guerrero (Fig. 3). This species has an estimated area of occupancy of ca. 36 km^2^ (criterion B1 < 500 km^2^) and has been recorded in four localities (condition a: ≤5 locations). The specimen from *Reyes 1064* (MEXU, UC/JEPS) from 1988 was collected in a cloud forest. The areas within 1 km of this locality have been actively transformed into crop fields (personal observations from 2019–2021), leading us to consider that condition b(iii) is appropriate for this case, which refers to a projected decline in area, extent, and quality of habitat. Therefore, following the guidelines to the IUCN criteria (IUCN 2022), *S.ibe-dzi* should be classified as Endangered [EN B1ab(iii)].

Being a hemiparasitic plant with a complete dependence on hosts, mistletoe populations are vulnerable to the indirect effects of logging important host trees such as oaks (*Quercus* spp.), and habitat modification and fragmentation for livestock and agriculture (Ávalos and Nixon 2004). In addition, most of the *Quercus* species from Mexico have not been evaluated to determine their conservation status (Valencia-Ávalos and Morales-Saldaña 2016). Press and Phoenix (2005) indicated that the local extinction of a preferred host may lead to population declines and subsequent extinctions of associated parasites, highlighting the importance of hosts for the long-term survival of mistletoes such as *S.ibe-dzi*, and the perpetuation of populations. Furthermore, parasitic plants have been historically stigmatized and have not received full attention in terms of conservation priorities, even though mistletoes are particularly sensitive to environmental stress and considered keystone species in forests (Watson 2001; Fontúrbel et al. 2018; Crates et al. 2022; Watson et al. 2022).

#### Notes.

**Figure 4.**
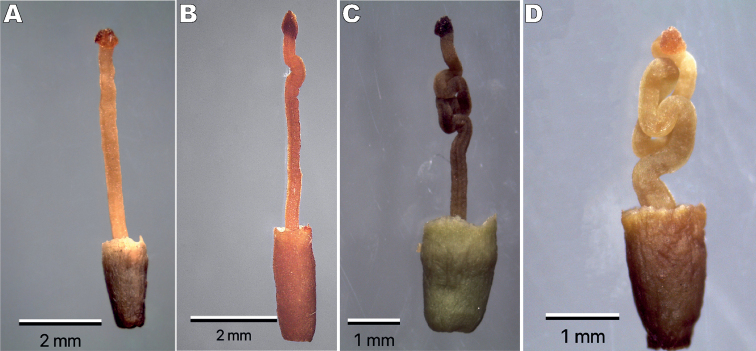
Style types in four species of *Struthanthus* (Loranthaceae) from Mexico **A***S.quercicola* (straight to sigmoid) **B***S.deppeanus* (sigmoid to slightly convoluted) **C***S.ibe-dzi* (convolute) **D***S.ramiro-cruzii* (strongly convolute).

Herbarium specimens of *S.ibe-dzi* have been previously identified as *S.deppeanus*, *S.quercicola*, or *Struthanthus* sp. However, the new taxon differs from *S.deppeanus* and *S.quercicola* by its compressed or bicarinate nodes and by having glaucous stems, leaves, and inflorescences which are covered by a whitish wax (observed in both fresh and dried specimens). In addition, *S.ibe-dzi* has one inflorescence raceme per axil, with peduncled triads with a sessile central flower and pedicellate lateral ones. The bracts and bracteoles are caducous, forming visible scars. Staminate flowers have asymmetrical thecae and an apiculate connectival horn in both series, while pistillate flowers have a convolute style with ± 3 longitudinal folds (Fig. 4C).

*Struthanthusibe-dzi*, as other mistletoes in the San Juan Mixtepec region of Oaxaca, are locally known as birdlime vine, and known for the sticky substance (i.e., viscin) produced in the fruits, which is used to catch birds (Hunn 2008).

### ﻿Key to *Struthanthusibe-dzi* and morphologically similar congeners

**Table:** 

1	All floral triads pedicellate; pedicels accrescent when bearing fruit	***S.deppeanus***
–	Lateral floral triads pedicellate or subpedicellate, central one sessile; pedicels not accrescent when bearing fruit	**2**
2	Bracts and bracteoles persistent, mainly in pistillate flowers; style straight to sigmoid in pistillate flowers	***S.quercicola***
–	Bracts and bracteoles caducous in both staminate and pistillate flowers; style convoluted to strongly convolute in pistillate flowers	**3**
3	Leaf base subcordate to truncate; staminate flowers 6–7 mm long; pistillate flowers 5.0–5.8 mm long	***S.ramiro-cruzii***
–	Leaf base cuneate to oblique; staminate flowers 6–9 mm long; pistillate flowers 5.8–7.2 mm long	***S.ibe-dzi***

## ﻿Discussion

Among the currently accepted genera of Loranthaceae, *Struthanthus* is one of the most taxonomically challenging since no monographic treatment exists (Kuijt 2016; Robles et al. 2016). Numerous specimens in herbaria lack reproductive structures, have immature pistillate or staminate inflorescences, or contain only fruits, raising difficulties regarding their classification. Moreover, the shape of leaf blades may vary within the same individual from young to old branches (Heide-Jørgensen 2008), posing additional identification challenges when specimens are scant or sterile. In fact, many species of *Struthanthus* have been described from specimens presenting only pistillate or staminate individuals, resulting in numerous synonyms in the genus (Caires and Dettke 2020). Also, specimens only collected with fruits are not always possible to separate from the related genus *Passovia* (Kuijt 2014). Therefore, fertile specimens from the same locality with both pistillate and staminate mature flowers are essential to facilitate identification of *Struthanthus* at both generic and species levels.

*Struthanthusibe-dzi* morphologically resembles *S.deppeanus*, *S.quercicola*, and *S.ramiro-cruzii*. All these four species have epicortical roots on stems and inflorescences in racemes. *Struthanthusdeppeanus*, *S.ibe-dzi*, and *S.quercicola* have similar leaf shape ranging from ovate to lanceolate with a long acute apex (Table 1). Pistillate mature flowers in *S.quercicola* (Fig. 4A) have a straight to sigmoid style, whereas it is sigmoid to slightly convolute in *S.deppeanus* (Fig. 4B), convolute in *S.ibe-dzi* (Fig. 4C), and strongly convolute in *S.ramiro-cruzii* (Fig. 4D). These stylar convolutions have been previously described for other Mexican species of *Struthanthus* and *Cladocolea* (Kuijt 1975a, b, 2003b, 2009a, 2014; Kuijt and Hansen 2015) as well as in three species of *Peristethium* and *Struthanthus* from Ecuador and Peru: *P.polystachyum* (Ruiz & Pav.) Kuijt, *P.tortistylum* (Kuijt) Kuijt, and *S.ophiostylus* Kuijt (Kuijt 2012, 2014). Besides the above-described characters, these four species of *Struthanthus* present geographical structure by being reported for the province of Sierra Madre del Sur. However, *S.deppeanus* and *S.quercicola* are also widely distributed across Mexico and Central America, while *S.ibe-dzi* and *S.ramiro-cruzii* are restricted to this province (Fig. 3). In terms of elevational ranges, *S.deppeanus* occurs at elevations from 950 to 2,900 m a.s.l., *S.ibe-dzi* from 1,960 to 2,650 m a.s.l., *S.quercicola* from 1,100 to 2,100 m a.s.l., and *S.ramiro-cruzii* is distributed from 1,600 to 2,200 m a.s.l.

**Table 1.** Comparison of morphological characters among four species of *Struthanthus* (Loranthaceae) from Mexico: *S.deppeanus*, *S.ibe-dzi*, *S.quercicola*, and *S.ramiro-cruzii*.

Character	*S.ibe-dzi*	*S.deppeanus*	*S.quercicola*	*S.ramiro-cruzii*
Stem nodes	compressed and bicarinate	terete	terete	terete
Leaf shape	ovate to lanceolate, rare elliptical	lanceolate to ovate-lanceolate	lanceolate to elliptic-lanceolate	ovate to lanceolate, cordate when mature
Leaf apex shape	acute to acuminate	acute to long acuminate	attenuate to acuminate	apiculate or acute
Leaf base shape	cuneate to oblique	acute	obtuse to round	subcordate to truncate
Petiole length (mm)	2.5–12	5–10	3–8	5–10
Inflorescences per axil	1	1–2	1–2	1 (2)
Triads per staminate inflorescence	6–16 (19)	8–10	8–16	10–12
Triads per pistillate inflorescence	6–12 (15)	8–10	6–12	8–14
Bracteoles persistence	caducous	caducous	persistent	caducous
Pistillate flower length from base of ovary (mm)	5.8–7.2	6.2–7.5	4.0–5.2	5.0–5.8
Style	convolute	sigmoid to slightly convoluted below the stigma	straight to sigmoid	strongly convolute
Staminate flower length from base of ovary (mm)	6–9	7.0–8.5	5.5–7	6–7

## Supplementary Material

XML Treatment for Struthanthus ibe-dzi
